# Detecting Rare Variants in Case-Parents Association Studies

**DOI:** 10.1371/journal.pone.0074310

**Published:** 2013-09-26

**Authors:** Kuang-Fu Cheng, Jin-Hua Chen

**Affiliations:** 1 Biostatistics Center and Department of Epidemiology, Taipei Medical University, Taipei, Taiwan; 2 Graduate Institute of Statistics, National Central University, Chungli, Taiwan; National Taiwan University, Taiwan

## Abstract

Despite the success of genome-wide association studies (GWASs) in detecting common variants (minor allele frequency ≥0.05) many suggested that rare variants also contribute to the genetic architecture of diseases. Recently, researchers demonstrated that rare variants can show a strong stratification which may not be corrected by using existing methods. In this paper, we focus on a case-parents study and consider methods for testing group-wise association between multiple rare (and common) variants in a gene region and a disease. All tests depend on the numbers of transmitted mutant alleles from parents to their diseased children across variants and hence they are robust to the effect of population stratification. We use extensive simulation studies to compare the performance of four competing tests: the largest single-variant transmission disequilibrium test (*TDT*), multivariable test, combined *TDT*, and a likelihood ratio test based on a random-effects model. We find that the likelihood ratio test is most powerful in a wide range of settings and there is no negative impact to its power performance when common variants are also included in the analysis. If deleterious and protective variants are simultaneously analyzed, the likelihood ratio test was generally insensitive to the effect directionality, unless the effects are extremely inconsistent in one direction.

## Introduction

In human medical genetics, one often hypothesizes that genetic susceptibility to common diseases such as diabetes is mainly due to the alleles that have moderate frequencies in the population. However, there is increasing evidence showing that there is extreme allelic and locus heterogeneity and multiple rare variants underlie susceptibility to such diseases. [1], [2] So far, GWASs, focusing mainly on common single nucleotide polymorphisms (SNPs), have detected over 2000 loci associated with diseases and traits [3]. However, many identified SNPs have very small effect sizes and the proportion of heritability explained by common variants is only modest [4]. Recently, Cirulli and Goldstein [5] evaluated the evidence for an important role of rare variants of major effect in common diseases and suggested the hypothesis that multiple rare gene variants, each with moderate to high penetrance, could play an important role in common diseases [6]–[8]. Some studies have demonstrated that both common and rare alleles may lead to the same disease. For examples, multiple rare mutations were found to be related to early-onset Alzheimer’s disease [9]. Rare mutations in genes involved in immune response also confer a high risk for lupus [10]. An effective way to discover disease-associated rare variants is through direct sequencing of relevant regions (for examples, linkage regions, all exons, all promoters). Botstein and Risch [11] suggested study of nonsynonymous SNPs in common diseases. With the advances in resequencing technologies, many believed that it is possible to search systematically for rare variant effects that are not tagged by panels of common SNPs.

In detecting associations with common variants, two approaches are often used. One approach is the single-variant test with family-wise error rate controlled by a multiple testing correction (for example, Bonferroni, permutation). Another approach is to use a multivariable method for testing all variants simultaneously. However, application of either approach with multiple rare variants involves multiplicity (that is, large degrees of freedom or large number of comparisons) and data sparseness, which will reduce power. To solve this problem when multiple rare variants are expected to jointly influence disease risk, an often-used approach is to group the variants according to some identified function and to test the combined effect of multiple rare variants. This motivates several collapsing methods, with or without weighting, to enrich association signals and at the same time reduce degrees of freedom [12]–[16].

Collapsing methods were mainly designed for detecting rare variants in case-control studies. However, recently, Mathieson and McVean [17] showed that the bias due to rare variant population stratification was typically stronger than the bias due to common variant population stratification and existing association tests for case-control studies may not be able to correct for its effect. In this paper, methods for analyzing sequence data from case-parents studies are investigated. The related methods for detecting single variant association are well known to be robust to the effect of population stratification**.** In the case of detecting association with multiple rare variants, we show these methods continue to be robust.

Here, we focus on a case-parents scenario in which a group of rare variants has been identified. Our aim is to test whether these rare variants are associated with the disease of interest. Four tests are considered for detecting rare variants. The first test is based on the largest single-variant *TDT* [18]. The second test is a multivariable test proposed by Zhang et al. [19]. Their approach uses a score vector based on the differences of transmitted and non-transmitted genotypic codes across all variants. The third test is a simple extension of the *TDT* based on combining the numbers of transmitted mutant alleles across all variants from parents, called combined TDT (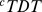). The fourth test is a novel method derived from the use of a random-effects model. The p-values of the four tests are computed using a permutation argument. By using this approach, we only need genotype data of the variants for computing the four test statistics. All tests discussed here depend on the number of the transmitted mutant alleles from heterozygous parents. Thus they are robust to the effect of population stratification. We investigate the validity of the four tests and their power performance under various conditions on the number of families, number of functional variants and their effect sizes, number of nonfunctional variants in the region, variant frequency and effect directionality. The simulation results indicated that for non-functional gene, the type I errors of the tests all could be adequately controlled near to the designated significance level, if our permutation method was used. The results also concluded that the test based on the random-effects model was most powerful under all conditions used in simulations. We found no negative effect to its power performance when rare and common variants were analyzed simultaneously. In addition, the impact of effect directionality was small and particularly so when the number of family trios became large.

## Materials and Methods

In this section, we give a genetic model for family data under allelic heterogeneity. We briefly describe the single-variant *TDT* and multivariable test, and give a formal definition of the combined *TDT*. Lastly, we propose a new test based on a random-effects model with a mixture distribution. Simulations are used for empirical evaluation of type I error rates and power.

### Genetic Model and Basic Results

We assume that within a gene region, there are 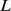 variants that may cause disease susceptibility. Here region is referred to the unit in which the variants are collectively analyzed. For each variant, we choose which allele of the variant to consider as mutation. Usually, this will be the minor allele. We focus discussion on a family study with sample of 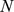 family trios. It is possible to extend our statistics for nuclear families or even general pedigrees [20]. However, a common feature shared by common and rare variants is that they tend to give rise to a weak familial concentration of cases [6].

At each variant site, we assume that genotypes of father, mother and a diseased child were observed across families. Let genotypes 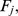 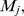 and 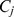 denote the number of mutant alleles carried by father, mother, and the diseased child, respectively, at the 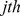 variant site. The minor allele frequency (MAF) of the 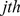 variant is denoted by 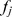. For genotype 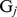 at the 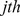 variant site, let 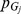 denote the penentrance of the genotype. The genotype relative risks (RRs) at the 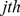 variant site are defined as 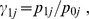 and
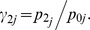



We assume Hardy-Weinberg Equilibrium (HWE) holds. Thus 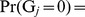 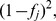 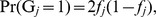 and 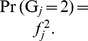 The usual TDT is calculated under three mating types: mating type 2, if 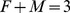, mating type 4, if 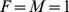, and mating type 5, if 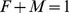 [21], where at least one of the parents is heterozygous. For the 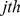 variant, the probability that the parent is heterozygous and transmits the mutant allele, conditional on the offspring having the disease, is given by
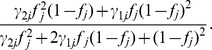



The same probability but not transmitting the mutant allele is given by
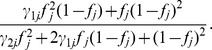



Thus, given the heterozygous parent with respect to the 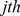 variant (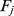 = 1), the probability of transmitting a mutant allele to the diseased offspring is given by
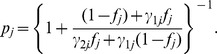



Under the null association, this probability equals to 1/2. In this paper, we focus our discussion on the dominant genetic model (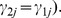 We point out that since the rare variants have very low allele frequencies, the chance of a subject carrying two mutant alleles is small. Thus, the power of the proposed association tests under the recessive model tends to be small. However, the corresponding power under other genetic models is expected to be similar to that under the dominant genetic model.

### Single-variant *TDT*

One approach of association studies is to test the association of each variant separately using a univariable test and assess the significance of the overall test after correction for multiple comparisons. The *TDT* is the most popular univariable test in family studies. It compares the numbers of transmitted mutant alleles and non-transmitted mutant alleles from heterozygous parents of the diseased children. Specifically, for the 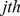 variant, we denote the number of transmitted mutant alleles from heterozygous parents as 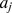 and the number of non-transmitted mutant alleles as 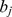. The *TDT* statistic is defined by 
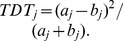
 If the overall type I error rate of the multiple testing is determined at 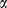 level, and Bonferroni correction is used, then the significance level for each single-marker test should be 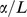. Unfortunately, this approach tends to have conservative type I error and is under-powered in general. To mitigate this problem, one could use the test statistic 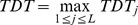 and apply the following permutation method to compute its p-value. This permutation method consists of n permutation steps. In the *ith* permutation step, the transmitted and non-transmitted genotype vectors within each family are randomly permuted and the test statistic 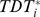 based the *ith* permuted data set is computed. The p-value is defined as the proportion of times that 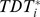 exceeds 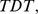 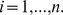 This test procedure is denoted as 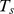.

### Multivariable Test

A multivariable test for the study of association is to test all variants simultaneously using a multivariable technique. Here, we focus on the approach suggested by Zhang et al. [19]. A very similar multi-marker method was also proposed by Shi, et al. [22]. Let genotypes 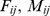 and 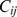 denote the number of mutant alleles carried by father, mother, and the diseased child, respectively, in the 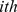 family at the 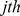 variant site. Zhang et al. considered the difference of the numbers of transmitted and non-transmitted mutant alleles 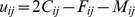 and multiple-marker scores 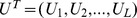 where 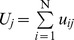 (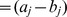). The variance-covariance estimate of the scores under the null hypothesis was given by 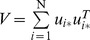, where 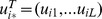. The statistic of the test was defined as 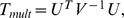 where 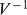 is the generalized inverse of 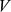. Under the null hypothesis of no association, 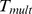 has asymptotic 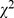 distribution with degrees of freedom equal to the rank of 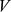. However, the p-value based on the asymptotic result also gives conservative type I error and low power, unless the number of markers is small. In this paper, we propose using the same permutation method described above to compute its p-value.

### Combined *TDT*

Another approach based on the transmitted genotypes is the collapsing method. This method is to combine transmitted (non-transmitted) mutant alleles, usually minor alleles, across all variants to enrich the signals of mutation and simultaneously reduce the degrees of freedom. Several versions of the combining method also can be considered in this regard. However, one simple and yet more powerful version is to combine the numbers of transmitted and non-transmitted mutant alleles across all variants by defining 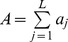 and 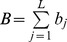. The combined *TDT* statistic is defined by 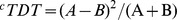.

Under the null hypothesis, the asymptotic distribution of 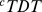 is not totally clear, since the variants may be correlated. Here, we also propose using the same permutation method to compute its p-value.

### Likelihood Ratio Test Based on a Random-effects Model

At the *jth* variant, we note that 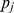 is the probability of a heterozygous parent transmitting the mutant allele to his/her affected offspring, and 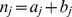 is the total number of heterozygous parents in the sample. Thus, conditional on 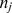 heterozygous parents, the probability of observing 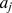 mutant alleles transmitted is given by
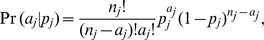
a binomial probability, if there is no population stratification. However, in the case of no linkage and no association association, 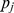  = 0.5 and the probability 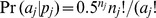 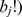 is a binomial probability, even there is population stratification. Thus, any test, depending only on the numbers of transmitted and non-transmitted alleles, has type I error robust to the population stratification.

We note that 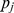 is bounded, 0.5< 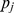 <1, if the variant is functional (defined to be risk-related, here and after) and has deleterious effect. Very often, however, one can identify an upper bound for relative risks, say 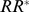. Under this situation and a dominant genetic model, the transmission probabilities is bounded by 0.5 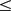 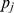 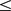 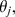 where 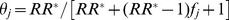 is treated as a known quantity. Here 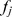 is the estimate of allele frequency based on the case-parents genotypes under HWE condition [23]. We let the proportion of the functional variants in the gene region be given by 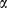, 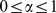, and follow the formulation of random-effects model (Laird and Ware [24]) to treat 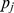 as a random variable. Then 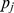 has a mixture distribution given by 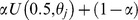 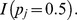 Here, 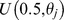 is a uniform distribution over the interval between 0.5 and 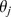, and 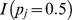 is a distribution with probability mass 1 at 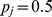. From this simple model one finds that association testing can be done by simply testing H_0_: 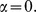 That is, the fraction of functional variants is zero. We note that the value of 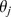 is inversely related to the magnitude of the MAF 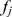. Thus according to the mixture distribution, the random effect 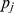 is assumed to take smaller mean effect value when the corresponding MAF is larger. This model property agrees with the usual observation in the genetic studies that common variants tend to have smaller effects and rare variants tend to have moderate or larger effects; see Smith and Lusis [25].

To infer parameter 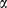 , we consider the marginal distribution of 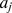. This can be done by combining the binomial distribution and mixture distribution. Simple calculation shows that the marginal distribution of 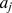 is given by
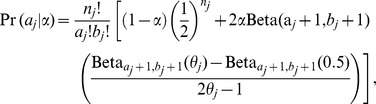
where 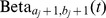 is the cdf of a beta distribution evaluated at 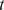 and has parameters (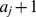,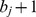) and 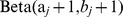 is the usual beta function evaluated at 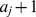 and 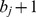. We use a pseudo likelihood function, defined by 
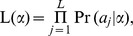
 to work as if it is the true likelihood. This is because that the numbers of transmitted alleles 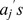 across all variants may not be independent. We also use the usual likelihood ratio statistic 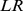  = 2log 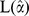
−2log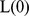 to test the null hypothesis, where 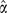 is the usual maximum likelihood estimate of 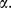 We suggest that the p-value of the likelihood ratio test (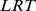) also be computed through the use of the same permutation method described above.

### Evaluation of Type I Error Rate and Power

To evaluate the performance of four tests: 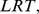 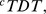 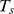 and 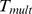, simulations were used to generate diplotypes of parents and diseased children for the 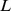 variants within a gene region. We first generated parental diplotypes. We used coalescent model [26] that mimics the LD pattern, recombination rate and population history for the Europeans to simulate realistic sequence-level genetic data. We randomly selected one gene region with 250 kb in length and used COSI [26] to generate a pool of 25000 haplotypes. From this pool of haplotypes, we calculated the haplotype frequencies of all variants and generated parental diplotypes. The diplotype of the offspring was determined by randomly transmitting one haplotype from each of his/her parents. The disease status of the offspring was determined by the relative risks and disease prevalence. Let 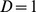 denote that the child is diseased and 0, otherwise. Also recall that 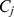 is the number of mutant alleles carried by the diseased child at the 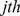 variant site. The relative risk (RR) model was given by
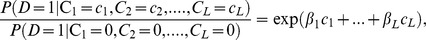
where the relative risk due to the 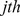 variant was 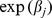. The RR of a functional variant was determined according to its 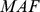 and the RR-range (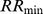 to 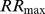) of all functional variants, through the following formula: 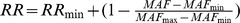 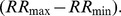 Here, 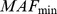
(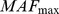) was the smallest (largest) MAF of all functional variants. This in effect means that functional variant with smaller frequency was assigned larger effect size in the simulations.

In all simulations, we randomly selected 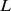 variants from the simulated region and assumed the first 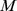 variants were functional and the rest were nonfunctional. In all scenarios, rare variants with minor allele frequencies (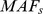) ranging from 0.1% to 1% were randomly selected. Higher frequency (common) variants had 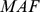 either 3% or 5%. Among the randomly selected rare variants in our simulations, the largest minor allele frequency (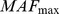) was 0.00936 and the smallest minor allele frequency (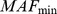) was 0.00120.

Given the disease risks for all functional variants, haplotype frequencies, and disease prevalence rate equal to 1%, we determined the disease status of each offspring and sampled families until a pre-specified number of case-parents trios (500–2000) was reached. In order to evaluate type I error rate of each test under the null hypothesis, we considered regions with (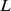 = ) 5–100 rare variants. Next, in order to study the power performance and its impact due to the inclusion of the nonfunctional variants in the studied region, we considered regions with (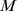 = ) 2 and 10 functional variants, and RR ranges 1.8–2.0 and 3.4–3.6, respectively. The percentage of nonfunctional variants was chosen to be 0%, 50%, 67% 75%, 80%, 90%, or 95%. The functional variants may be protective or deleterious. To study its impact, we also considered a region with 10 functional variants and each variant had 10%, 20%, 30%, or 40% chance of being protective. The RR of the protective variant was taken as the inverse of that for the deleterious variant. Finally, in order to study the power performance of the tests when rare and common variants in the same region were simultaneously analyzed, we considered a region with 5 functional variants and 20 nonfunctional variants under two scenarios: (1) functional variants included 1 or 2 common variants with 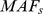 equal to 3%, 5% and the rest of the variants rare; (2) nonfunctional variants included 1 or 2 common variants with 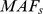 equal to 3%, 5%, and the rest of the variants rare.

All simulation results given in this paper were based on 5000 independently simulated samples. In each sample, we performed n = 10,000 permutation steps to compute p-values for all tests. The 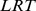 statistic was defined with the choice of relative risk upper bound 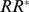 = 5. Type I error rates and power of all tests were estimated by the proportions of replicates with p-value <0.05.

## Results

### Empirical Type I Error Rates

Table 1 shows empirical type I error rates of the competing tests in detecting rare variants when the significance level was designed at 5%. From Table 1, we first found that the type I error rates of 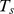 were slightly conservative when sample size was small. However, its performance can be improved by increasing the number of family trios. Under all simulated conditions, the range of its type I errors was (0.0325, 0.0592). In contrast, the corresponding type I error ranges for 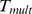, 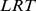 and 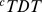 were respectively given by (0.0436, 0.0564), (0.0425,0.0580) and (0.0308, 0.0470). This indicates that the permutation method proposed in the paper can effectively control type I errors of all tests near the designated level.

**Table 1:** Type I error rates of the tests when the true significance level is 5%.

	5 nonfunctional variants				10 nonfunctional variants				20 nonfunctional variants			
Sample size	T_s_	T_mult_	LRT	^c^TDT	T_s_	T_mult_	LRT	^c^TDT	T_s_	T_mult_	LRT	^c^TDT
500	0.0328	0.0436	0.0496	0.0308	0.0378	0.0438	0.0470	0.0318	0.0325	0.0475	0.0425	0.0312
1000	0.0336	0.0440	0.0462	0.0380	0.0478	0.0518	0.0460	0.0420	0.0522	0.0548	0.0508	0.0438
2000	0.0452	0.0474	0.0558	0.0384	0.0592	0.0558	0.0504	0.0452	0.0438	0.0548	0.0452	0.0470
	**50 nonfunctional variants**				**100 nonfunctional variants**							
**Sample size**	**T_s_**	**T_mult_**	**LRT**	**^c^TDT**	**T_s_**	**T_mult_**	**LRT**	**^c^TDT**				
500	0.0372	0.0452	0.0450	0.0442	0.0342	0.0460	0.0510	0.0420				
1000	0.0414	0.0564	0.0580	0.0414	0.0464	0.0517	0.0568	0.0460				
2000	0.0514	0.0486	0.0482	0.0416	0.0544	0.0474	0.0498	0.0400				

### Empirical Power when Nonfunctional Variants were Included

Here, we considered the regions with 2 or 10 functional variants and 0%–95% nonfunctional variants. The effect sizes of the functional variants were inversely proportional to the number of functional variants. The results from Table 2 first indicated that the power of the test depends more on the effect size of the variant and less on the number of functional variants. For example, in the case of large effect size (RRs between 3.4 and 3.6) but a small number ( = 2) of functional variants, most of the power was greater than 80% when the family number was moderate or large (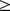
1000) and the proportion of the functional variants in the gene region was at least 10%. Under the same conditions, however, the power of the tests was often smaller than 70% when the effect size was small (RRs between 1.8 and 2.0), but the number of functional variants was large ( = 10). The power of the tests tended to be decreased when the proportion of nonfunctional variants was increased to create more noise. However, we found that the tests 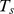 and 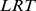 were less sensitive to the nonfunctional variants, and particularly so when the functional variants had larger effect sizes. Unfortunately, the power of 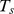 was too small in most cases. In contrast, 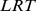 has better empirical power under all simulation conditions considered. The power loss due to the inclusion of nonfunctional variants can be reduced by increasing sample size. For example, if the number of family trios was increased to 2000, the power of the tests 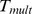 and 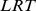 can be maintained above 80% under all conditions. If the family number was decreased to 500, 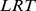 was still able to maintain its power ranging from 0.7426 to 0.9592 when the proportion of functional variants was no less than 50%. However, under the same condition, the corresponding range for the second best test was only between 0.6650 and 0.7948. Obviously, there was a substantial power advantage accrued by using 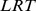.

**Table 2:** The power of the tests when nonfunctional rare variants are included.

		2 functional variants^a^				10 functional variants^b^			
Sample Size	Proportion offunctional variants	T_s_	T_mult_	LRT	^c^TDT	T_s_	T_mult_	LRT	^c^TDT
500	100%	0.8334	0.8896	0.9592	0.9152	0.2674	0.4606	0.8370	0.7948
	50%	0.8000	0.8376	0.9254	0.8146	0.2438	0.3412	0.7426	0.6650
	33%	0.7830	0.7778	0.8840	0.6946	0.2018	0.2776	0.6112	0.4642
	25%	0.7718	0.7150	0.8714	0.6594	0.1752	0.2270	0.4936	0.3230
	20%	0.7534	0.6792	0.8542	0.5978	0.1618	0.2182	0.4448	0.2756
	10%	0.7240	0.5516	0.7888	0.4376	0.1308	0.1542	0.3178	0.1842
	5%	0.6642	0.3696	0.6818	0.1698	0.1229	0.1114	0.2434	0.1280
1000	100%	0.9922	0.9960	0.9998	0.9986	0.5288	0.8168	0.9788	0.9864
	50%	0.9904	0.9874	0.9976	0.9896	0.4600	0.7034	0.9208	0.9268
	33%	0.9866	0.9802	0.9956	0.9608	0.4022	0.5990	0.8508	0.7776
	25%	0.9812	0.9662	0.9898	0.9304	0.3530	0.5108	0.7834	0.5870
	20%	0.9760	0.9522	0.9890	0.8890	0.3286	0.4568	0.7264	0.5142
	10%	0.9672	0.8754	0.9814	0.7312	0.2636	0.3190	0.5618	0.1867
	5%	0.9446	0.7406	0.9554	0.3164	0.2400	0.2241	0.4634	0.1789
2000	100%	1.0000	1.0000	1.0000	1.0000	0.8724	0.9886	0.9974	1.0000
	50%	1.0000	1.0000	1.0000	1.0000	0.8086	0.9718	0.9890	0.9990
	33%	1.0000	1.0000	1.0000	0.9992	0.7556	0.9474	0.9822	0.9788
	25%	1.0000	0.9994	1.0000	0.9980	0.7290	0.9000	0.9694	0.8932
	20%	1.0000	0.9994	1.0000	0.9948	0.6874	0.8616	0.9614	0.8237
	10%	1.0000	0.9974	1.0000	0.9574	0.6101	0.7133	0.8919	0.5888
	5%	1.0000	0.9850	1.0000	0.5662	0.5375	0.5392	0.8255	0.4189

a RRs are 3.4–3.6.

b RRs are 1.8–2.0.

### Power when the Effects were in Different Directions

Table 3 shows the power performance of four competing tests when the effects of the variants were in different directions. In the simulations, the probability of a functional variant having protective effect was assumed to be between 10% and 40%. Clearly, if the probability was 10%, then the effects were more consistent in one direction, and if the probability was 40%, then the effects were less consistent. We focus on a region with 10 functional variants. From the results of Table 3, we found that when sample size was small, the power of 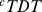 was more sensitive to the effect directionality. For example, when the family number was 1000, the power of 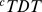 was decreased from 92.36% to 59.44% as the probability of protective variant was increased from 10% to 40%, while the power of 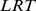 was decreased from 93.28% to 75.58%. When the family number was increased to 2000, the corresponding power was decreased from 99.72% to 87.78% and 99.40% to 95.58%, respectively. In the former case, the ranges for 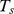 and 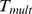 were 47.00%–51.08%, and 73.30%–80.30%, respectively. These results show that 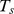 and 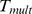 were more robust to the effect directionality. However, they are less powerful when the family number was small or moderate. Overall, we found that 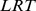 had the best power performance when the variants have different effect directions. However, in the case of a large number of family trios ( = 2000), 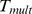 and 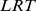 were found to be more comparable in power.

**Table 3:** The power of the tests when effects are in different directions^a^.

	Proportion of protective variants							
	10%				20%			
Sample size	T_s_	T_mult_	LRT	^c^TDT	T_s_	T_mult_	LRT	^c^TDT
500	0.2628	0.4380	0.7510	0.6480	0.2476	0.4198	0.6836	0.5582
1000	0.5108	0.8030	0.9328	0.9236	0.5096	0.7864	0.9132	0.8676
2000	0.8512	0.9888	0.9940	0.9972	0.8532	0.9870	0.9896	0.9918
	**30%**				**40%**			
**Sample size**	**T_s_**	**T_mult_**	**LRT**	**^c^TDT**	**T_s_**	**T_mult_**	**LRT**	**^c^TDT**
500	0.2576	0.4072	0.6102	0.4516	0.2388	0.3922	0.5040	0.3302
1000	0.5004	0.7676	0.8584	0.7870	0.4700	0.7330	0.7558	0.5944
2000	0.8284	0.9832	0.9842	0.9758	0.8194	0.9768	0.9558	0.8778

a Deleterious variants have RRs between 1.8 and 2.0. Protective variants have RRs between 0.50 and 0.56.

### Empirical Power when Common Variants were Included

Table 4 gives the power results when the common and rare variants were analyzed simultaneously. It is well known that both the magnitude of allele frequency and effect size of functional variant have impact on the power performance of test. From the results of Table 4 we found that in family study, low effect common variant tended to have greater positive impact in power than high effect rare variant. Although we have observed that 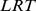 had the best power performance if all functional variants had low MAFs. We found that the power performance of all tests became more comparable when common but low effect variants were included in the study and the number of family trios was moderate or large (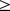 500). In the case of smaller number of family trios (300), however, their power differences became greater. In this case, 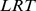 still had the best power performance. It had power 0.8629 and 0.9986, if one and two rare functional variants were replaced by common functional variants, respectively. The second best test was 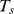 and its power was 0.6975 and 0.9806, respectively.

**Table 4:** The power of the tests when high frequency functional and nonfunctional variants were included^a^.

		Sample size							
		300				500			
Number of high frequency functional variants^b^	Number of low frequency functional variants^b^	T_s_	T_mult_	LRT	cTDT	T_s_	T_mult_	LRT	cTDT
0	5	0.2186	0.2182	0.4386	0.2353	0.3506	0.3230	0.6200	0.3568
1	4	0.6975	0.6030	0.8629	0.4868	0.9440	0.8532	0.9796	0.7156
2	3	0.9806	0.9530	0.9986	0.8558	1.0000	0.9995	1.0000	0.9814
		**Sample size**							
		**500**				**1000**			
**Number of high frequency non-functional variants** c	**Number of low frequency non-functional variants** c	**T_s_**	**T_mult_**	**LRT**	c **TDT**	**T_s_**	**T_mult_**	**LRT**	c **TDT**
0	20	0.3506	0.3230	0.6200	0.3568	0.6789	0.6708	0.8930	0.6641
1	19	0.3506	0.3230	0.6200	0.3568	0.6792	0.6706	0.8931	0.6642
2	18	0.3498	0.3214	0.6184	0.3558	0.6794	0.6708	0.8933	0.6641

a The first high frequency variant has MAF 3% and the second high frequency variant has MAF 5%. High frequency functional variants have RRs between 1.1 and 1.2. The low frequency variants have MAFs between 0.1% and 1% and low frequency functional variants have RRs between 2.4 and 2.6.

b There are 5 high or low frequency functional variants and is no nonfunctional variants.

c The total number of variants is 25. Among them, there are 20 high or low frequency nonfunctional variants and 5 low frequency functional variants.

When nonfunctional common variants replaced nonfunctional rare variants in the analysis to create more noise, we also found that 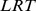 had better power performance. However, the power change of any test seemed small. For example, in the case of 500 family trios and 1 or 2 nonfunctional rare variants were replaced by nonfunctional common variants, the power range for 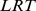 was (0.6184, 0.6200). The corresponding ranges for other tests were (0.3498, 0.3506), (0.3214, 0.3230), and (0.3558, 0.3568). The power range became even smaller if the number of family trios became larger. These results indicated that the tests were insensitive to the nonfunctional common variants.

## Discussion

There is emerging interest in association studies of rare variants and it is hypothesized that rare variants are more likely to be functional than common variants are. Although it is still not totally clear how exactly rare and common variants affect the disease, some results have shown that the combination of GWAS and sequencing could be a good technique for studying diseases [27]. GWAS is used for localizing signals to a small region of the genome and sequencing is applied to find and define the specific variations that may be underlying the causes that led to the signal. In this process, one powerful method for identifying rare variants is to pool variants by gene or pathway into a composite test. Most tests considered so far for detecting rare variants were based on population-based case-control studies. However, the method proposed for detecting rare variants in case-control studies needs extra effort to control for the potential confounding effect due to the population stratification. Li and Leal [12] suggested that the problem may be overcome by implementing logistic regression in which covariates for describing ethnicity are included in the analysis; also see Wu et al. [15] and Lin and Tang [16]. However, Mathieson and Mcvean [17] pointed out that the existing methods can fail to correct for rare variant stratification. They suggested that more robust approach with respect to stratification, such as family-based association, should be used for replication. In this paper, we have described four statistical methods for detecting group-wise association between multiple rare variants in a locus and a genetically heterogeneous disease, based on sequence data.

All tests discussed in this paper depend on the differences between the numbers of the transmitted and non-transmitted mutant alleles from heterozygous parents to their diseased children, across all variants and families. The properties of the tests depend only on the transmission probabilities of the mutant alleles from parents to their children, and consequently, they are robust to the effect of population stratification. In the simulations, we also have investigated the effect of the population stratification on type I error rate. We considered two populations, Europeans and Africa Americans, and used the coalescent model for the Africa Americans to generate sequence-level data at the same variant locations for the Europeans (as described in the simulation section), with 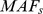 ranging from 0.001 to 0.03. The disease prevalence rate and the proportion of the second population were set at 4% and 20%, respectively. Unreported results showed that all type I errors were between 0.04 and 0.06 and all tests were truly robust to the effect of population stratification.

Although the tests considered depend only on the transmitted and non-transmitted genotype vectors, they are defined in different ways or use different concepts. The combined *TDT* is defined to enrich the association signals across variants, while the single-variant and multivariable tests are not. The likelihood ratio test based on a random-effect model is a novel test derived from the use of a completely different concept. It is designed to test zero proportion of functional variants in a gene region. Other tests were designed to compare the genotype frequencies of the “case” and “control” populations. We have compared the performance of the four tests: 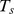, 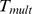, 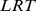 and 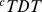. From our limited simulation study, we found that if the number of family trios is small (500), 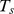 and 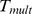 seems to be less powerful when the region size (that is, L) is larger, and 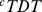 tends to have poorer power performance when the region size is smaller. Whether this is in general true, more study is needed. If the number of family trios is increased, however, the power differences of all tests become very small unless the proportion of functional variants in the gene region is very small. Overall, under any simulation condition given in this paper, we find that 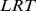 has the best power performance.

In our computation of p-values, a permutation method based on random permutation of the transmitted and non-transmitted genotype vectors within each family is used; Shi et al. [22] also used the same technique. One advantage of using this permutation approach is that haplotype data are not required. We need genotype data only. The results from the null simulations indicate that this approach can effectively control type I errors near to the designated 5% significance level.

A number of studies have shown that alleles with a wide range of frequencies are involved in disease etiology [28]–[36]. Some simulation results showed that the collapsing methods for detecting rare variants in case-control studies could be adversely influenced by the presence of low-effect common variants (see for example Li and Leal [12]). In this paper, we also have compared the impacts of low-effect (RRs between 1.2 and 1.4) common variants (MAFs
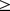
3%) and high-effect (RRs between 2.4 and 2.6) rare variants (MAFs between 0.1% and 1%) in family studies. Our simulated results suggested that the low-effect common variants have greater positive impact in power than the high-effect rare variants. We emphasize that this finding is observed under the given sample size, RRs and MAFs, chosen for comparison in simulations. Same simulation study also showed that all tests were very robust to the inclusion of nonfunctional common variants in the analysis. In general, we find no negative impact produced by analyzing rare and common variants simultaneously in a family study.

The disadvantage of the usual collapsing methods is that they are not powerful when the effects are not all in the same direction. We also have investigated the properties of the competing tests when the effects are in different directions. In general, if the proportion of the variants that are protective is increased, then the power of the tests is decreased. However, if the number of family trios is increased, then the power loss can be reduced. 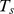 and 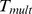 are more robust in the sense that their percentages of power loss are smaller. Unfortunately, they are less powerful. The power loss of 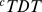 is the largest. Thus one should be careful when it is used for detecting rare variants. Finally, we observe that although 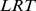 is not the most robust test, but it is the most powerful test in all cases. Thus this method can be recommended for detecting rare variants.

The definition of the combined *TDT* is based on the equal weighting strategy. Madsen and Browning [14] proposed a strategy to weight each variant according to allele frequencies in case-control study. Their idea is to assign lower weights for the common variants. However, our simulation results suggested that the tests considered here were robust to the inclusion of nonfunctional common variants and the power can be improved if functional common variant was included in the analysis with the same weight as that for rare variant. Price et al. [13] suggested a variable-threshold approach to demonstrate that the incorporation of computational predictions of the functional effects of missense variants can provide improvement in power. We note that a weighting strategy that can adapt to properties of individual variants may be applied to improve power too. For example, in our random-effect model approach, different effect distributions were used for variants with different MAFs. According to the distribution property, the range (from 0.5 to 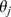) of the random effect was smaller for variants with larger MAFs and larger for variants with smaller MAFs. If we know more precisely the range of RRs, we also can use a more precise upper bound for RRs and hence a better random-effects model. This in general can lead to the power improvement of the likelihood ratio test. In our simulations, we have used RR upper bound equal 5 and the results seems acceptable. Thus, unless one has more information so that smaller upper bound can be used, otherwise, this upper bound is recommended.

Note that the family study requires genotypes of the diseased individuals and their parents. Unfortunately, parental genotypes may not be always available. Several methods had been proposed to solve the issue of missing parental genotype, for example, using EM algorithm with the assumption that genotype missing is random conditional on the known genotypes of other family members; see the discussion in Chen and Cheng [37]. These methods are difficult to extend to the cases of multiple variants with very small allele frequencies. For some diseases it might be easy to obtain the genotype of one parent either because the other parent is not available for study or he/she refuses to participate. In such circumstances, one may follow the idea of 1-*TDT* (Sun et al. [38]) for example to utilize genotype information for diseased individuals and only one available parent for each diseased individual. Another alternative approach is to compare variant genotypes in affected and unaffected offspring, instead of using variant data from parents; see for examples, Spielman and Ewens [39], Siegmund, et al. [40], Lunetta, et al. [41], and Zhao et al. [42]. Note that we would require more complicated method for computing p-values for the approach by Sun et al. [38]. On the other hand, for the alternative approach, a within family permutation argument may be sufficient. The detailed methods and results will also be reported elsewhere.

In this paper, we report only the simulation results under the multiplicative genetic model. The unreported power under the dominant genetic models only differs slightly but basic conclusions remain unchanged. One major reason is that the subject cases have only very small chance of being homozygous for the mutant allele, because of low MAF. For the same reason, we also find that all competing tests have very small power in detecting rare variants under the recessive genetic model.
